# Superhydrophobic ZnO networks with high water adhesion

**DOI:** 10.1186/1556-276X-9-385

**Published:** 2014-08-08

**Authors:** Camelia Florica, Nicoleta Preda, Monica Enculescu, Irina Zgura, Marcela Socol, Ionut Enculescu

**Affiliations:** 1 National Institute of Materials Physics, P.O. Box MG-7, Magurele, Bucharest 077125, Romania

**Keywords:** ZnO rod networks, Chemical bath deposition, Ammonia, Superhydrophobicity, High water adhesion

## Abstract

ZnO structures were deposited using a simple chemical bath deposition technique onto interdigitated electrodes fabricated by a conventional photolithography method on SiO_2_/Si substrates. The X-ray diffraction studies show that the ZnO samples have a hexagonal wurtzite crystalline structure. The scanning electron microscopy observations prove that the substrates are uniformly covered by ZnO networks formed by monodisperse rods. The ZnO rod average diameter and length were tuned by controlling reactants' concentration and reaction time. Optical spectroscopy measurements demonstrate that all the samples display bandgap values and emission bands typical for ZnO. The electrical measurements reveal percolating networks which are highly sensitive when the samples are exposed to ammonia vapors, a variation in their resistance with the exposure time being evidenced. Other important characteristics are that the ZnO rod networks exhibit superhydrophobicity, with water contact angles exceeding 150° and a high water droplet adhesion. Reproducible, easily scalable, and low-cost chemical bath deposition and photolithography techniques could provide a facile approach to fabricate such ZnO networks and devices based on them for a wide range of applications where multifunctionality, i.e., sensing and superhydrophobicity, properties are required.

**PACS:**

81.07.-b; 81.05.Dz; 68.08.Bc

## Background

Zinc oxide, a semiconductor characterized by a direct bandgap (3.37 eV), a large exciton binding energy (60 meV), and a high transmittance of visible light [1], can be easily engineered to yield functionalities based on its outstanding optical and electrical properties [2-6]. Moreover, as a metal oxide with, probably, the richest family of structural morphology including whiskers, wires, rods, tubes, belts, cages, rings, combs, prisms, etc. [6-11], ZnO may achieve new properties and become a technological key material, its nanostructures representing an interesting choice for the fabrication of electronic and optoelectronic micro/nanodevices. Furthermore, morphology influences other properties such as wettability, another significant characteristic of ZnO-covered surfaces bringing great advantages in a wide variety of applications [12-15]. Recently, special attention has been paid to superhydrophobic ZnO surfaces with high water adhesion [16-18]. The polymorphic properties of ZnO low-dimensional structures triggered different functionalities and therefore enabled different applications. This led to an increased interest in developing new ZnO synthesis methods by various physical (pulsed laser deposition, molecular beam epitaxy, chemical vapor deposition, magnetron sputtering, thermal evaporation) and chemical (chemical bath deposition, electrochemical deposition, hydrothermal, solvothermal, sol-gel, precipitation) techniques [19-24]. Compared to the physical route where harsh conditions such as high temperature or special equipments are usually required and consequently generating high costs, the solution-based chemical approach presents several advantages including the following: easily accessible raw materials, the use of inexpensive equipment, scalability, and control of the morphologies and properties of the final products by changing different experimental parameters. When using low-cost and highly efficient methods, like chemical bath deposition for obtaining desired morphologies, the preparation technique is more and more attractive for mass production.

When designing electronic or optoelectronic micro/nanodevices based on ZnO, a patterning technique such as electron-beam lithography or photolithography is combined with a ZnO preparation method, e.g., hydrothermal growth or chemical bath deposition in order to achieve functionality [25-29]. Photolithography is a conventional patterning approach representing a highly efficient and cost-effective technique of producing metallic electrodes, yielding large patterned surfaces in a short time. On the other hand, the chemical bath deposition is a versatile deposition method with the following main advantages: relatively low process temperature (below 100°C), ambient pressure processing, and the use of inexpensive equipments.

In the present paper, this simple and inexpensive solution process was used to grow ZnO rods quasi-monodispersed in size on Au-patterned SiO_2_/Si substrate obtained by photolithography. The influence of the reaction parameters, such as reactants' concentration and reaction time, on the morphological, structural, and optical properties of the ZnO rods was studied using scanning electron microscopy, X-ray diffraction, optical spectroscopy, and photoluminescence. In addition, the electrical and the wetting properties of ZnO network rods were investigated. Because the interdigitated electrodes are connected by ZnO networks forming different junctions, no additional lithographical steps are necessary for contacting them. By using two-probe current-voltage measurements, a variation of the ZnO sample resistance was evidenced when these samples were exposed to ammonia. Finally, a superhydrophobic behavior with high water adhesion was observed for all samples regardless of the rod dimensions. Such properties are very helpful for designing devices for sensors, open microfluidic devices based on high adhesive superhydrophobic surface implying no loss of microdroplet reversible transportation [30], or micro total analysis systems by their synergetic combination.

## Methods

**Figure 1:**
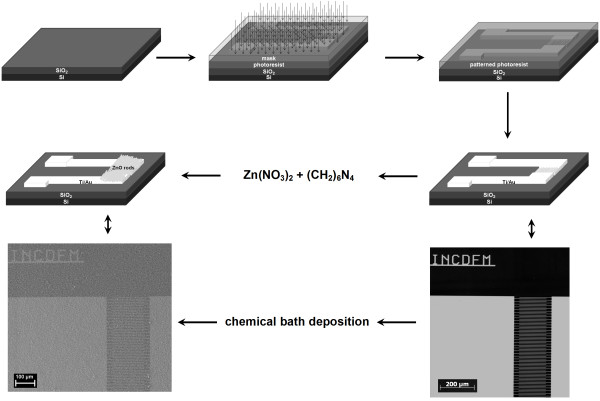
**Schematic illustration of the experimental procedures.** Schematic illustration of the experimental procedures involved in the preparation of interdigitated metallic electrodes by photolithography technique, further used in the growth of ZnO network structures by chemical bath deposition.

Initially, a typical standard photolithographic resist patterning step was used in order to create the metallic interdigitated electrode structures. Thus, a photoresist (AZ 5214E, MicroChemicals, Ulm, Germany) was spin coated on the SiO_2_/Si substrate, and by thermal treatments and UV light exposures in subsequent steps through a mask, the interdigitated electrodes were formed on a 0.4-mm^2^-size area having a width of 4 μm and gaps of 4 μm. Further, after the developing procedure, in the sputtering/evaporation step, a 10-nm Ti layer is required before the deposition of a 90-nm Au layer for the improvement of the gold adhesion on the SiO_2_/Si substrate. After removing the photoresist in acetone by a lift-off procedure, the metallic interdigitated electrodes are ready to use for the ZnO preparation by chemical bath deposition. Thus, the substrates containing the finger grid structures were immersed in a beaker containing aqueous solutions of zinc nitrate (Zn(NO_3_)_2_, Sigma-Aldrich, St. Louis, MO, USA) and hexamethylenetetramine ((CH_2_)_6_N_4_, Sigma-Aldrich) of equal molarities (0.05, 0.1, or 0.2 mM). The beaker was sealed and heated at a constant temperature of 90°. Two deposition times (3 and 6 h) were used. Finally, the samples were removed from the solution, rinsed with distilled water, and dried at room temperature. A schematic representation of the photolithographic and deposition steps is depicted in Figure 1.

According to [31], the ZnO synthesis by chemical bath deposition involves the following chemical reactions:

Zn(NO_3_)_2_ → Zn^2+^ + 2NO_3_^-^(a)

(CH_2_)_6_N_4_ + 6H_2_O → 6HCHO + 4NH_3_(b)

NH_3_ + H_2_O → NH_4_^+^ + HO^-^(c)

Zn^2+^ + 3NH_4_^+^ → [Zn(NH_3_)_4_]^2+^(d)

[Zn(NH_3_)_4_]^2+^ + 2HO^-^ → Zn(OH)_2_ + 4NH_3_(e)

Zn(OH)_2_ → ZnO + H_2_O(f)

The exact function of the (CH_2_)_6_N_4_ in the ZnO synthesis is still unclear. As a non-ionic cyclic tertiary amine, it can act as a bidentate Lewis ligand capable of bridging two Zn^2+^ ions in solution [32]. Moreover, it has been suggested [33] that it is preferentially attached to the non-polar facets of the ZnO crystallite cutting off the access of zinc ions towards those facets, favoring the polar (001) face for growth. (CH_2_)_6_N_4_ is also known as a weak base and pH buffer [34], being considered a steady source for slow release of HO^−^ ions. All these (CH_2_)_6_N_4_ characteristics influence the nucleation and the growth rates of different ZnO crystal facets, processes responsible for the overall structure and morphology. We investigate the dependence of the ZnO morphology for different reaction parameters varying the precursors' concentration (both reactants with 0.05, 0.1, or 0.2 mM, the Zn(NO_3_)_2_/(CH_2_)_6_N_4_ molar ratio was always 1:1) and the deposition time (3 and 6 h). Thus, the synthesized samples were labeled as follows: *a* (0.05 mM, 3 h), *b* (0.1 mM, 3 h), *c* (0.2 mM, 3 h), *d* (0.05 mM, 6 h), *e* (0.1 mM, 6 h), and *f* (0.2 mM, 6 h).

The crystalline phase of the samples was identified by X-ray diffraction (XRD) on a Bruker AXS D8 Advance instrument (Karlsruhe, Germany) with Cu Kα radiation (*λ* = 0.154 nm). The source was operated at 40 kV and 40 mA and the Kα radiation was removed using a nickel filter.

The optical properties of the ZnO samples were investigated by measuring the total reflection spectra using a PerkinElmer Lambda 45 UV-VIS spectrophotometer (Waltham, MA, USA) equipped with an integrating sphere. The photoluminescence (PL) measurements were performed at 350 nm excitation wavelength using FL 920 Edinburgh Instruments spectrometer (Livingston, UK) with a 450-W Xe lamp excitation and double monochromators on both excitation and emission. All PL spectra were recorded in the same experimental conditions (excitation wavelength = 350 nm, step, dwell time, slits).

The sample morphologies were evaluated using a Zeiss Evo 50 XVP scanning electron microscope (SEM, Oberkochen, Germany).

Electrical measurements were carried out using a Keithley 4200 SCS (Cleveland, OH, USA) and a Cascade Microtech MPS 150 probe station (Thiendorf, Germany). The current-voltage characteristics were obtained by the conventional two-probe method on the samples exposed at different times and at room temperature to ammonia vapors (an area of about 3 mm^2^ in size contains the patterned metallic stripes and millimeter-sized electrodes).

The wetting properties of the ZnO samples were determined by measuring the static contact angle (CA) with a Drop Shape Analysis System, model DSA100 from Kruss GmbH (Hamburg, Germany) [35]. The sample was placed on a plane stage, under the tip of a water-dispensing disposable blunt-end stainless steel needle with an outer diameter of 0.5 mm. The needle was attached to a syringe pump controlled by a PC for delivery of the water droplet to the test surface. Drop volume was gradually increased until the drop adhered to the surface this being achieved when the volume reached approximately 3 to 4 μl. All the CA measurements were carried out in the static regime at room temperature. The analysis of the CA and of other drop parameters were performed by the PC using the DSA3® software supplied with the instrument. CA was measured by fitting a circle equation to the shape of the sessile drop (due to the sphere-like shape of the drop) and then calculating the slope of the tangent to the drop at the liquid-solid vapor interface line. The camera was positioned in order to observe the droplet under an angle of about 2° to 4° with respect to the plane of the sample surface supporting the droplet. Roll-off angles were measured with a goniometer in order to control the tilt angle. The orthoscopic images were obtained using a commercial photocamera.

## Results and discussion

**Figure 2:**
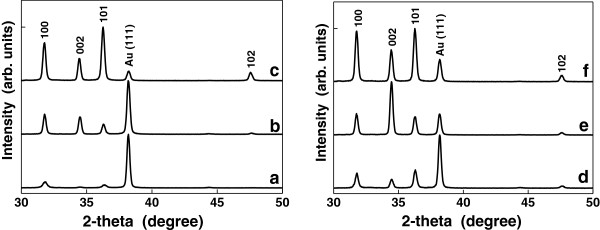
The XRD patterns of all ZnO samples.

The samples' structure was examined by X-ray diffraction, the XRD patterns being presented in Figure 2. Four peaks can be readily indexed to hexagonal wurtzite ZnO (JCPDS file no. 36-1451) corresponding to the Miller indexes of the reflecting planes for ZnO (100), (002), (101), and (102). The strong and sharp diffraction peaks suggest that the as-obtained products are well crystallized. Interestingly, the intensity distribution of some XRD peaks deviates drastically from what is characteristic to standard ZnO where (101) is the strongest XRD line and the intensity ratio [*I*(002)/*I*(101)] = 0.56 is the value for non-preferred orientation. For example, in the case of sample *b* and sample *e*, the intensity ratio [*I*(002)/*I*(101)] increases, its values larger than 1 being correlated with a high degree of orientation on the *c*-axis of the ZnO crystallites. The peak at 2*θ* = 38.3° is assigned to Au (111). With the increase of the reactants' concentration and the reaction time, the peak intensity corresponding to gold decreases, suggesting a better covering of the substrate.

**Figure 3:**
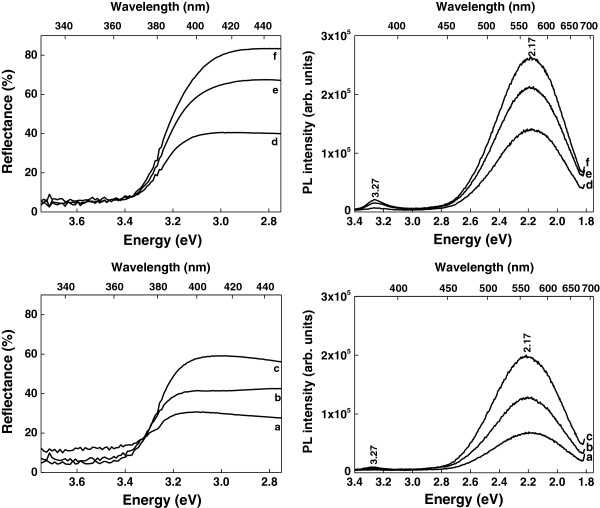
Reflectance and photoluminescence spectra of all ZnO samples.

The room temperature reflectance and photoluminescence (PL) spectra of the synthesized samples are shown in Figure 3. A strong decrease of reflectance can be noticed at approximately 380 nm in all sample spectra, this being attributed to the band-to-band transition in ZnO. Indeed, the bandgap value was estimated at around 3.27 eV by using the Kubelka-Munk function *F*(*R*) = (1 – *R*)^1/2^/2*R*, *R* being the observed diffuse reflectance. The PL spectra exhibit a strong, broad emission band centered at about 550 nm (2.17 eV) and a weak (or very weak) emission band centered at about 380 nm (3.27 eV). The UV emission has an excitonic origin, being attributed to the recombination of free excitons. Usually, the green emission is linked to some defects, being related to the incorporation of hydroxyl groups in the crystal lattice during the growth process and to the oxygen defects (interstitial ions or vacancies) [36-39]. Due to the fact that when employing wet chemical methods the ZnO crystallites are formed by Zn(OH)_2_ dehydration, traces of this compound on the ZnO surface lead to the quenching of the ZnO exciton emission [40]. Consequently, we may say that the optical properties of our ZnO samples are typical for this semiconductor.

**Figure 4:**
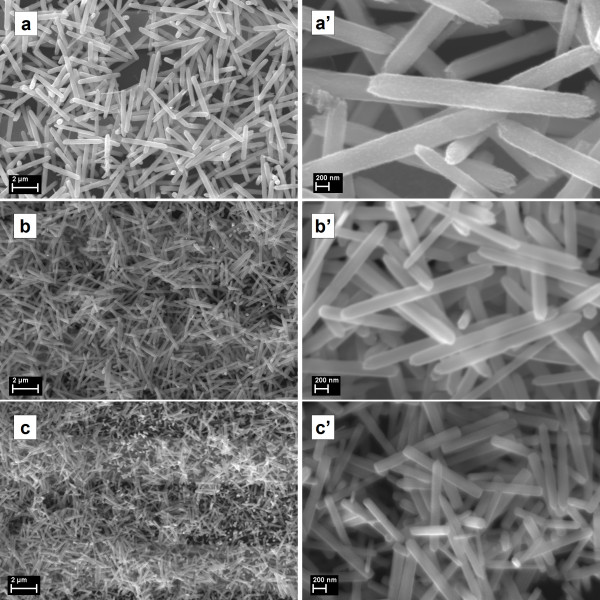
**SEM images of ZnO samples obtained at 3 h deposition time (also at higher magnification). (a**, **b**, **c)** SEM images of ZnO samples obtained at 3 h deposition time. **(a**′, **b**′, **c**′**)** The higher-magnification SEM images for the corresponding samples are also presented.

**Figure 5:**
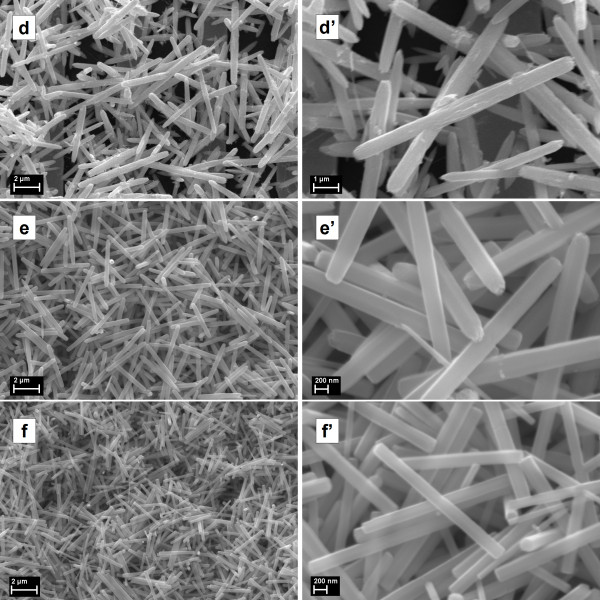
**SEM images of ZnO samples obtained at 6 h deposition time (also at higher magnification). (d**, **e**, **f)** SEM images of ZnO samples obtained at 6 h deposition time. **(d**′, **e**′, **f**′**)** The higher-magnification SEM images for the corresponding samples are also presented.

Further, the sample morphology was investigated by SEM (Figures 4 and 5). It can be seen that the samples exhibit random network structures formed by rods with relatively uniform dimensions (diameter (D) and length (L)) depending on the initial reaction parameters. From the higher-magnification SEM images the following (D, L) values for ZnO rod were estimated: sample *a* (350 nm, 3.5 μm), sample *b* (220 nm, 2.3 μm), sample *c* (170 nm, 1.4 μm), sample *d* (800 nm, 8 μm), sample *e* (340 nm, 3.5 μm), and sample *f* (230 nm, 2.7 μm). In all cases, ZnO samples are characterized by a quasi-monodisperse distribution in size and an apparent diameter/length ratio of about 1/10. Higher reactant concentrations lead to a decrease of the ZnO rod size. In addition, the increase of the precursors' concentration results in an increase of the ZnO rod density. Although there are many studies reported in the literature about the aqueous solution growth of ZnO rods synthesized using as reactants Zn(NO_3_)_2_ and (CH_2_)_6_N_4_[22-24,32-34], a complete understanding of the growth mechanism has not yet been achieved. When (CH_2_)_6_N_4_ is added in the reaction bath, initially ammonia and formaldehyde are produced by its thermal decomposition. From the zinc nitrate hydrolysis, zinc ions are generated, which interact with ammonia forming [Zn(NH_3_)_4_]^2+^ complexes. Under heating, these complexes are decomposed and release Zn^2+^ and HO^−^ ions into solution, which subsequently lead to the formation of Zn(OH)_2_, which is further thermally dehydrated to ZnO. Regarding our experiments, in order to propose a nucleation-growth model, we should take into account that regardless of the reaction parameters, for all cases, size-quasi-monodispersed rods are obtained. Thus, it should be assumed that all the ZnO nuclei are formed approximately at the same moment after the reaction starts, in a precisely defined nucleation phase. Further, the growth phase takes place with similar rates on all the nuclei without any new nucleation sites on the substrate. Hence, the precursors' concentration is directly linked to the number of initial nuclei; for a lower concentration, we deal with a smaller number of ZnO nuclei, whereas a higher concentration is responsible for a larger number of ZnO nuclei, this hypothesis being sustained by direct SEM observation (Figures 4 and 5). Additionally, more nuclei lead to more growth sites and consequently producing ZnO rods with smaller dimensions, whereas fewer nuclei, i.e., fewer growth sites, favor the growth of ZnO rods with higher dimensions. Therefore, the precursors' concentrations determine the number of initial ZnO nuclei and can be linked to the ZnO rods' density and dimensions (diameter and length).

**Figure 6:**
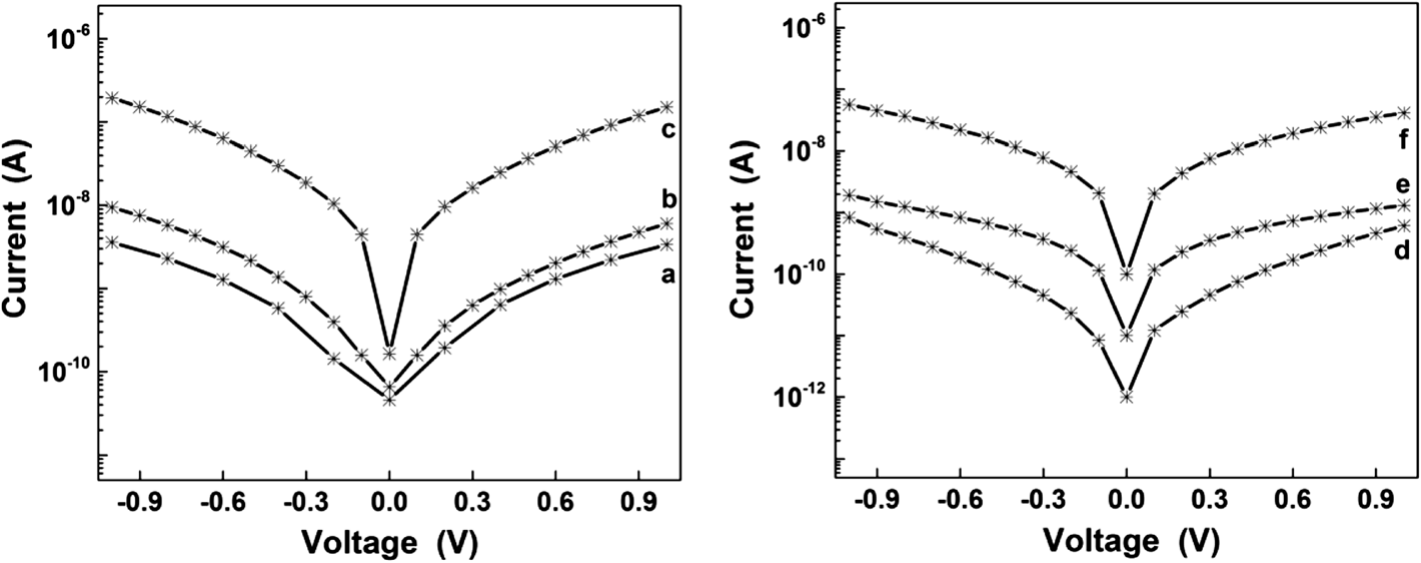
**The *I*-*V *** **characteristics of all ZnO samples.**

Notably, the deposited ZnO rods provide electrical paths between the neighboring finger grid structures. The network of ZnO rods covers both the patterned electrodes and the gaps between them, the electrical circuit being closed without a need for further steps. In Figure 6, plots of the current-voltage (*I*-*V*) characteristics measured in air are presented. The electric active area of the ZnO rods is 0.4 mm^2^. Since the resistance of the metallic fingers is less than 1 Ω, it can be neglected when discussing the samples' measured resistance, which originates from the deposited ZnO. The growth conditions of the ZnO network of rods are influencing the current values for each of the investigated sample. As it can be seen in the higher-magnification SEM images (Figures 4 and 5), the ZnO rods are in contact with each other, forming different types of junctions, like point, cross, or block junctions [41]. The electron transport throughout the network takes place by percolation through these junctions. The electrical properties of the investigated samples depend on the concentration of free electrons in the conduction band, which can be changed by oxidation or reduction reactions at the surface of the rods. This type of response is distinctive for n-type semiconductors [42,43]. While measuring in air, the atmospheric oxygen is adsorbed on the ZnO surface. The adsorbed oxygen can extract electrons available for conduction and become O_2_^−^, O^−^, or O^2−^[44].

**Figure 7:**
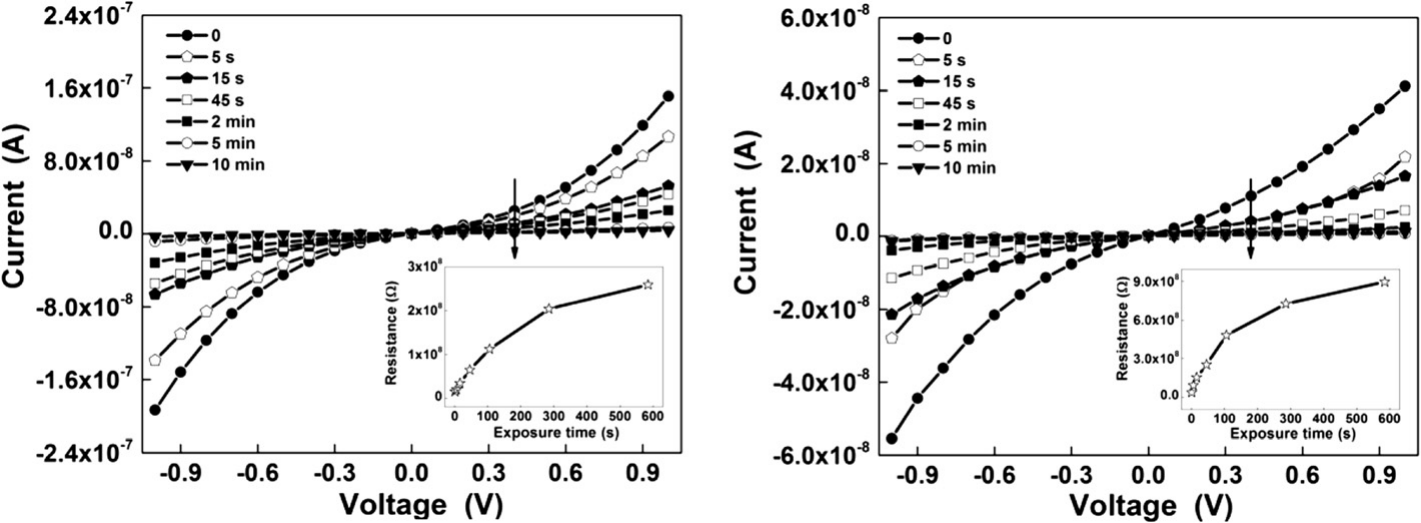
**Changes induced by exposure to ammonia in the current–voltage characteristics of ZnO networks.** Changes induced by exposure to ammonia in the current–voltage characteristics of ZnO networks on two representative samples: *c* (left) and *f* (right).

In order to reveal potential sensing applications for the ZnO networks deposited on interdigitated electrodes, an exposure to ammonia of two samples with higher values for current, sample *c* and sample *f*, was employed. In Figure 7, one can notice the differences in current and therefore in resistance when exposing the samples to ammonia for different times. In the insets are shown the resistance increases after the exposure to ammonia. Thus, sample *c* (Figure 7, left) has shown a resistance of 15 MΩ at 0.4 V in air. With ammonia exposure time, the resistance increased up to 20 MΩ (after 5 s), 112 MΩ (after 2 min), and 260 MΩ (after 10 min) at the same voltage. For sample *f* (Figure 7, right), the resistance was 36 MΩ at 0.4 V in air. The same increase in resistance was noticed with exposure time: up to 92 MΩ (after 5 s), 483 MΩ (after 2 min), and 900 MΩ (after 10 min). An increase in resistance was previously reported in literature when ZnO nanorods [43] or ZnO films [45] were exposed to ammonia. According with these references, the resistance increase can be linked to the adsorption processes which take place on the surface of the ZnO rods, either the direct absorption of ammonia or an enhanced absorption of atmospheric oxygen. The oxygen species described above and mentioned in [44] can be also responsible for the increase in resistance. The exposure to ammonia can enhance the adsorption of oxygen or water molecules to a certain extent, leading to a resistance increase, but the exact mechanism is still not explained. The saturation of the resistance occurs probably due to the saturation of the absorption processes which were favored by the presence of ammonia.

**Figure 8:**
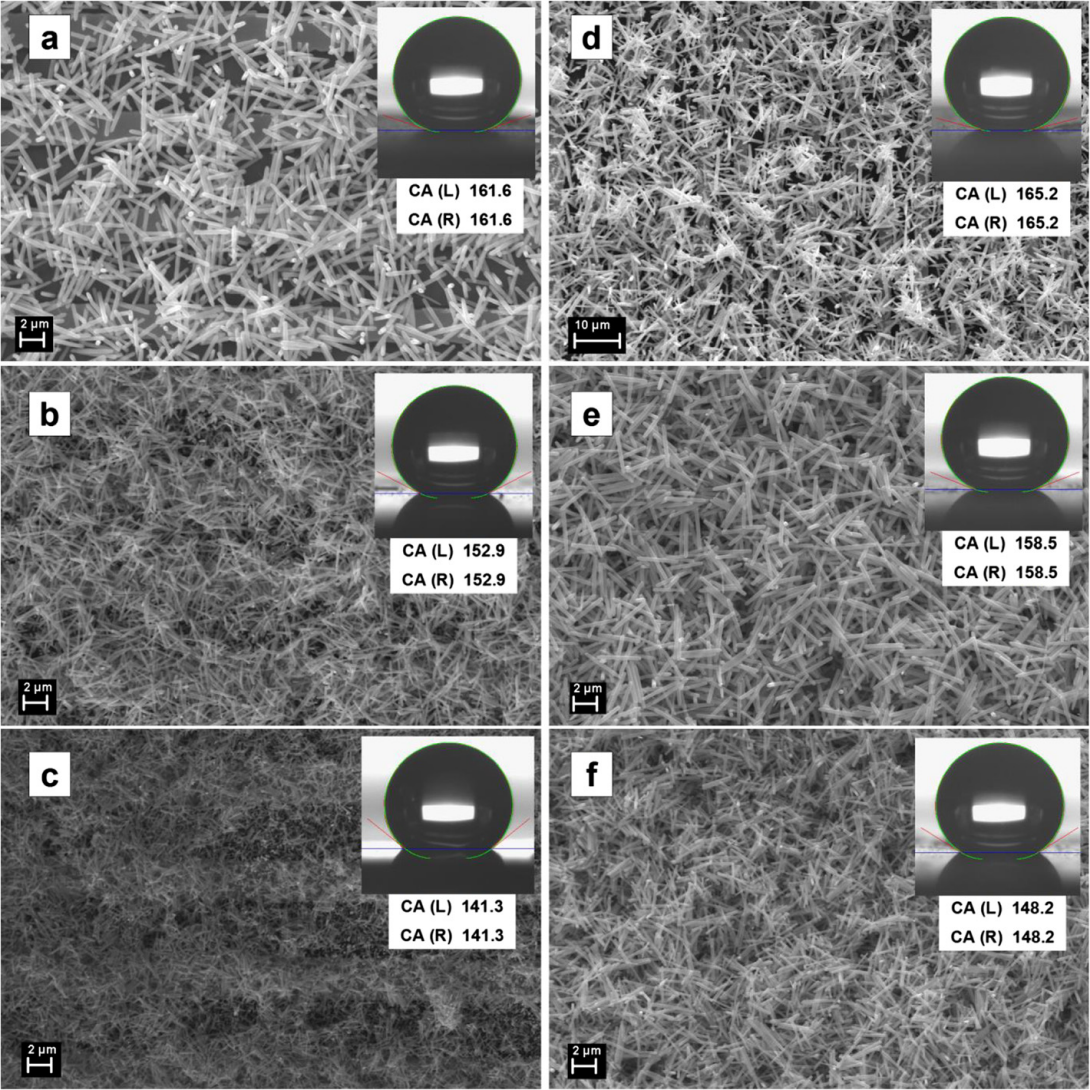
**SEM images and corresponding water droplet shapes images with CA values (insets) for ZnO samples.** SEM images for all ZnO samples obtained at 3 h **(a**-**c)** and 6 h **(d**-**f)** deposition time and their corresponding optical micrographs of the water droplet shape with the contact angle values shown in the insets, proving the superhydrophobic character of the ZnO networks.

Because such ZnO networks are formed by quasi-monodispersed rods, they can involve a large amount of trapped air in the empty spaces between individual structures leading to water-repellent properties. So, contact angle (CA) measurements were carried out for evaluating the wetting properties of such structures, the photographs of water droplets and corresponding SEM images being given in Figure 8. Thus, it is observed that all ZnO samples show hydrophobic (CA values above 140°) and even superhydrophobic (CA values exceeding 150°) behavior. In order to explain these results, we used the Cassie-Baxter relation in the form cosθ^*^ = *ϕ*_*S*_(cos*θ*_*E*_ + 1) − 1 [46], where θ^*^ is the CA formed on ZnO networks, *θ*_*E*_ is the CA formed on metallic pattern substrates (CA = 77°), and *ϕ*_*S*_ parameter is the fraction of the surface in contact with the water droplet. In the present case, the values of *ϕ*_*S*_ were obtained in the 0.03 to 0.2 domain for all samples. Based on these small values, the wetting behavior can be understood using the Cassie-Baxter model: the water droplet does not penetrate between the rods; it sits on a surface composed from both the ZnO network rods and the large amount of air bubbles included in the 3D interlaced structure, conferring, in this way, a highly water-repellent property. Practically, the air acts as a support ‘buffer’ for the water droplet which is in contact to the surface only in few small nanometric sites. Also, the *ϕ*_*S*_ values obtained for sample *d* (few rods with higher sizes) and for sample *c* (many rods with smaller sizes), 0.03 and 0.2, respectively, confirm that the spaces between rods depend on the rod dimensions influencing the CA values. The wetting properties are consistent with the electrical behavior, a higher quantity of the entrapped air resulting in a higher CA value and at the same time in a lower electrical resistivity. Thus, the samples' electrical resistance increases or decreases according to the density and individual properties of the rods covering the surface.

**Figure 9:**
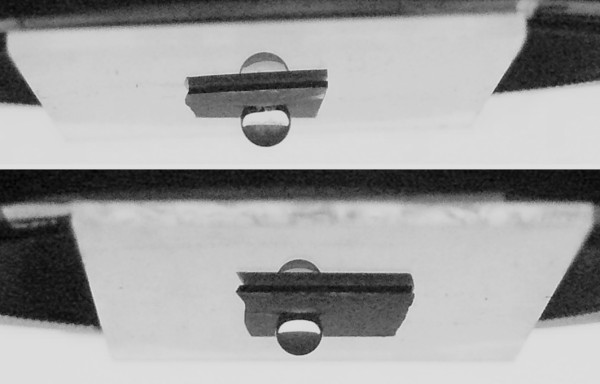
**Optical photographs of water droplet sitting on ZnO network samples vertically tilted.** Optical photographs of water droplet sitting on ZnO networks on two representative samples: *d* (up) and *c* (down) vertically tilted.

It is found that the water droplet does not slide when the substrate containing the ZnO networks is tilted to a vertical position or even turned upside down (Figure 9), resting stick, firmly pinned on the sample surface. The as-prepared ZnO network rod surface can hold 15 to 20 μl of a water droplet as a maximum quantity, which indicates an ultrastrong adhesive effect between the water droplet and the ZnO surface. Sample *d* (Figure 9, up) featured by the higher CA value (165°) is the sample which can sustain the biggest water volume suspended (20 μl) on its surface, responsible for the effect being numerous air pockets trapped between the ZnO rods characterized by the highest length and diameter values. When a water droplet exceeds 15 to 20 μl, gravity overcomes the adhesion force of the ZnO rod surface and the water droplet starts sliding.

Generally, such high adhesion between a water droplet and a superhydrophobic surface is explained considering the mechanism of the gecko's ability to climb up rapidly smooth, vertical surfaces. Each hair of the gecko's foot produces just a miniscule force through van der Waals' interactions, but millions of hairs collectively create the formidable adhesion [47]. In the present case, the ZnO structure-covered superhydrophobic surface is capable of making close contact with water droplets due to large van der Waals' forces, similar to the effect of the gecko's foot hairs. The high adhesive ability of such a superhydrophobic surface can be applied as a ‘mechanical hand’ in small water droplet transportation without any loss or contamination for microsample analysis [48-51].

## Conclusions

Random networks of ZnO rods can be obtained by combining a simple wet chemical route, i.e., chemical bath deposition, with a conventional patterning technique, photolithography. The ZnO rods show a hexagonal wurtzite structure and optical signatures (bandgap value and emission bands) typical for this semiconductor and method of synthesis. The electrical measurements revealed that the ZnO samples can exhibit interesting properties useful for chemical sensing. The contact angle measurements confirm that ZnO structure-covered surfaces present superhydrophobicity, with water contact angles exceeding 150° and a high water droplet adhesion, water volume suspended reaching 20 μl. Such superhydrophobic ZnO rod networks with high water-adhesive force have potential applications in no-loss liquid transportation. Moreover, because of the favorable surface to volume ratio of the 3D interlaced ZnO rods, they are expected to also have potential applications in gas sensors. Therefore, the results described herein regarding multifunctionality of ZnO-covered substrates are of great interest taking into account that the two methods used in sample preparation, chemical bath deposition and photolithography, are low cost and easily scalable, being efficient and suitable techniques for industrial processing.

## Abbreviations

CA: contact angle; D: diameter; L: length; PL: photoluminescence; SEM: scanning electron microscope; XRD: X-ray diffraction.

## Competing interests

The authors declare that they have no competing interests.

## Authors' contributions

CF designed the metallic interdigitated electrode structures, performed and analyzed the electrical measurements, and drafted the manuscript. NP designed the entire study, carried out the chemical bath deposition of ZnO rods, performed the optical measurements, and drafted the manuscript. ME performed the SEM measurements and made the corrections of the manuscript. IZ mainly helped to carry out the CA and roll-off angle measurements. MS helped with the analysis of XRD data. IE supervised the research, giving valuable advices about the whole experiments and manuscript. All authors read and approved the final manuscript.
